# Electrodeposition of Pb and PbO_2_ on Graphite Felt in Membraneless Flow-Through Reactor: A Method to Prepare Lightweight Electrode Grids for Lead-Acid Batteries

**DOI:** 10.3390/ma14206122

**Published:** 2021-10-15

**Authors:** Arminas Ilginis, Nerita Žmuidzinavičienė, Egidijus Griškonis

**Affiliations:** Department of Physical and Inorganic Chemistry, Faculty of Chemical Technology, Kaunas University of Technology, Radvilėnų pl. 19, 50254 Kaunas, Lithuania; nerita.zmuidzinaviciene@ktu.lt (N.Ž.); egidijus.griskonis@ktu.lt (E.G.)

**Keywords:** lead-acid batteries, graphite felt, electrodeposition, lightweight electrodes, flow-through reactor, composite electrode

## Abstract

One of the possible ways of mitigating the primary lead-acid battery downside—mass— is to replace the heavy lead grids that can add up to half of the total electrode’s mass. The grids can be exchanged for a lightweight, chemically inert, and conductive material such as graphite felt. To reduce carbon surface area, Pb/PbO_2_ can be electrochemically deposited on graphite felt. A flow-through reactor was applied to enhance penetration of adequate coverage of graphite felt fibers. Three types of electrolytes (acetate, nitrate, and methanesulfonate) and two additives (ligninsulfonate and Triton X-100) were tested. The prepared composite electrodes showed greater mechanical strength, up to 5 times lower electrical resistivity, and acted as Pb and PbO_2_ electrodes in sulfuric acid electrolytes.

## 1. Introduction

Lead-acid battery (LAB) is one of the most mature electrochemical energy storage technologies [1] and has been used for automotive applications for over 100 years. LABs are still popular due to robust electrochemistry, high recyclability, and low price [2], but significantly lag behind in terms of energy density and charge acceptance [3]. However, with increasing demand for hybrid and electric vehicles, requirements for automotive batteries have also escalated. Now, batteries need to be able to accept high rates of charge during braking of a vehicle, be lighter than ever, and work for many (at least 4000) cycles at a partial state of charge [4,5]. To accommodate LABs for hybrid/electric vehicles, researchers have introduced increased amounts of carbon additives to negative active mass. These graphitic or carbon materials can increase charge acceptance, reduce sulfation, and improve performance at a partial state of charge. The exact improvements depend considerably on the properties of the carbon additive and the amounts added to the electrochemically active paste [6,7]. Furthermore, there are suggestions to replace the negative LAB electrode partially or completely with a graphite electrode, which could act as a capacitor in high charge scenarios at a cost of reduced capacity [8]. The final application of carbon materials for LAB is substituting the traditional heavy lead grids for lightweight carbon/graphite sheets, foams, honeycomb, etc. [9,10,11]. These composite electrodes promise reduced weight and higher charge acceptance; however, self-discharge and parasitic hydrogen evolution reactions have been observed when increased amounts of carbon additives have been used in a negative electrode [12]. Electrodeposition of a thin layer of lead or its alloy on reticulated vitreous carbon from agitated methanesulfonate electrolyte has been proposed to reduce the downsides mentioned previously [13]. Alternative electrolytes can be used to deposit lead, such as acetate [14] and nitrate [15]. To increase the quality of the deposited layer, an additive can be used, such as sodium ligninsulfonate or Triton X-100 [16].

Graphite felt (GF) is an excellent material for the production of composite electrodes because it is lightweight, chemically resistant, has good electrical conductivity, and has sufficient mechanical strength [17]. Graphite felt, in comparison with carbon felt, has been chosen due to smaller electrical resistivity, which can be up to 6 times lower depending on the orientation of the measurement [18]. GF electrodes are widely used in electrochemistry, e.g., in flow-through batteries [19], fuel cells [20], and catalyst support [21]. However, it has not yet been investigated as a potential substituent of a high-density lead grid electrodes and current collector for LAB. Albeit various GF electrochemical modifications reported in the literature, electrodeposition of a uniform lead layer throughout the depth of GF is difficult due to the non-homogeneous distribution of the potential, favoring material deposition on the outermost fibers. One of the possibilities to increase homogeneity is to use a flow-through regime in a reactor with anodic and cathodic processes separated by an ionic exchange membrane [22].

In this study, a simplified flow-through reactor without proton exchange membrane membranes was proposed to produce composite GF-Pb and GF-PbO_2_ electrodes to be used in lead-acid batteries as lightweight current collectors. The morphology, electrochemical activity, and mechanical strength of the produced composite electrode were analyzed.

## 2. Materials and Methods

**Figure 1:**
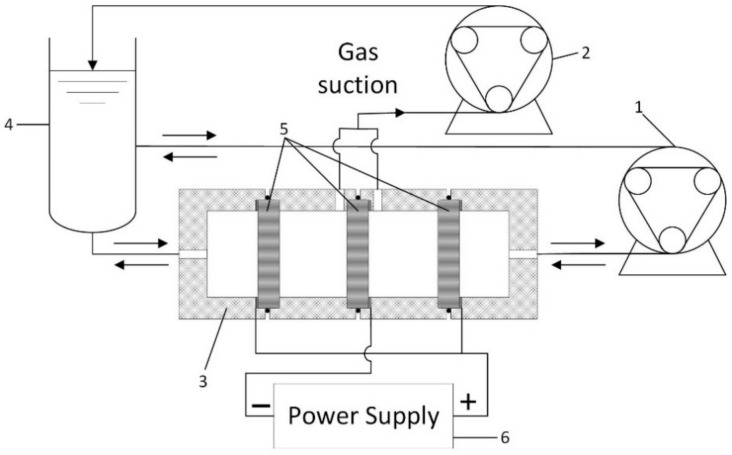
Electrodeposition flow-through reactor scheme: 1—main peristaltic pump for reversible electrolyte flow, 2—secondary peristaltic pump for gas evacuation, 3—PTFE reactor body, 4—electrolyte tank, 5—graphite felt electrodes, and 6—power supply.

Circular electrodes (50 mm diameter) for the flow-through reactor were cut from a GF sheet of 4.3 mm thickness (Wale Apparatus, Hellertown, PA, USA). The reactor used in this study (Figure 1) was custom made for this application. It consisted of 4 machined pieces of PTFE with rubber O-rings in between for sealing and 3 GF electrodes with a working area of 20 cm^2^ each—a cathode in the middle with anodes on both sides. Platinum wire was used for each electrode to ensure excellent electrical contact.

A system with two peristaltic pumps was applied to ensure zero gas interference on the electrode surface during electrochemical deposition. The main pump provided reversible flow through the GF electrodes at a constant rate of 120 mL min^−1^, whereas the secondary pump at a constant rate of 60 mL min^−1^ evacuated gas bubbles from around the cathode (Figure 1). In this system, flow direction was changed every 5 min of electrodeposition time by reversing the main peristaltic pump.

Three types of electrolytes (Table 1) were chosen without and with additives Triton X-100 (Sigma Aldrich, Saint Louis, MI, USA) or sodium ligninsulfonate (Tokyo Chemical Industry, Tokyo, Japan). Lead(II) nitrate (puriss. p.a., Reahim, Samara, Russia), nitric acid (an. gr., Reachem, Bratislava, Slovakia), lead(II) acetate trihydrate (puriss. p.a., Reahim, Samara, Russia), acetic acid (an. gr, Lach-Ner, Neratovice, Czech Republic), ammonium acetate (puriss. p.a., Reahim, Samara, Russia), lead(II) oxide (puriss. p.a., VEB Laborchemie Apolda, Apolda, Germany) and methanesulfonic acid (an. gr, Sigma Aldrich, Saint Louis, MI, USA) were used to prepare the electrolytes. The pH of the prepared electrolytes was measured using “Knick Portamess^®^ 910” (Knick Elektronische Messgeräte, Berlin, Germany) pH meter with “WTW SenTix 41” (Wissenschaftlich-Technische Werkstätten, Weilheim, Germany) electrode.

Prior to electrochemical deposition, 20% (by volume) isopropanol (Lachema, Brno, Czech Republic) solution was circulated through the reactor to wet the GF. After pumping out the isopropanol solution, 300 mL of distilled water was passed through the reactor to ensure all isopropanol was washed away. Then, after the removal of distilled water, the reactor was filled with corresponding electrolytes (about 200 mL). Electrochemical depositions were carried out under potentiostatic conditions at 2.5 V for 1 h. The current was recorded during the deposition by measuring a voltage drop across a known resistivity resistor, which was then converted to current using Ohm’s Law. The potential drop across the resistor was recorded using the “PicoLog TC-04” (Pico Technology, St Neots, UK) data logger. The total charge that passed through the cell was calculated from the recorded data.

**Figure 2:**
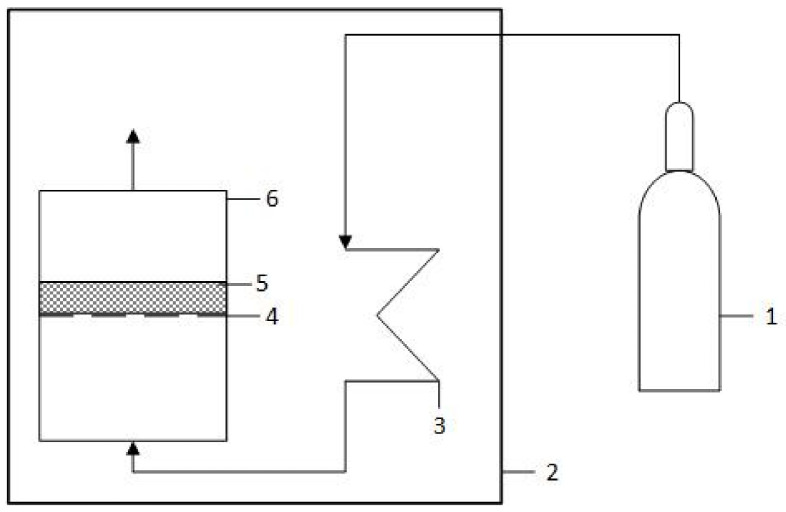
Scheme for drying of a sample under nitrogen atmosphere: 1—nitrogen gas cylinder, 2—heated chamber, 3—copper coil for gas heating, 4—mesh, 5—modified GF sample, and 6—aluminum drying container.

After deposition, the reactor was evacuated off the electrolyte and then washed by circulating 400 mL of distilled water twice. Then, cathodes and anodes were taken out of the reactor and dried under airless conditions in a special container (Figure 2), which had a constant flow of 1 L min^−1^ nitrogen gas (99.996%, Linde), at 60 °C for 2 h. These conditions are particularly important when drying cathodes to prevent oxidation of electrodeposited metallic lead that occurs rapidly at elevated temperatures and high humidity [23]. Completely dried samples were weighed using analytical balances ACJ (Kern, Balingen, Germany).

X-ray diffraction (XRD) analysis of samples after electrodeposition was carried out on “Bruker D8 Advance” (Bruker Corporation, Billerica, MA, USA). Machine settings were CuKα radiation and Ni filter by using 0.02° steps that measure intensity for 0.5 s in the range from 10.0° to 67.5°.

Morphology studies were carried out with scanning electron microscopy (SEM). Images were captured using “Hitachi S-3400N” (Hitachi Group, Tokyo, Japan) at 1000× magnification. Electron beam acceleration voltage was set to 15 kV.

For electrochemical tests, bare and modified samples of GF were placed in a polycarbonate sample holder, which limited the electrode area to 0.125 cm^2^. Platinum wire was used to provide an electrical connection for the samples. Electrochemical analysis was carried out using potentiostat–galvanostat “BioLogic SAS SP-150” (Biolgic, Seyssinet-Pariset, France) with EC-Lab ^®^ v10.39 software. 38% sulfuric acid electrolyte was used for cyclic voltammetry tests. The experiments were recorded at a sweep rate of 5 mVs^−1^ with a potential range of −0.8 to 0 V vs. saturated silver chloride electrode for samples polarized negatively during electrodeposition and 1.0 to 2.2 V vs. Ag/AgCl for samples polarized positively.

Electrical resistivity tests were performed using the same potentiostat–galvanostat BioLogic SAS SP-150 using silver-plated clamps (resistance of which was measured separately) for contact with the samples. The sample’s width was 7 mm and the distance between the clamps was 15 mm. The resistance of the samples was calculated using a linear fit tool on data from the linear sweep experiments. The circuit and clamp resistance was deducted from obtained resistance values to produce only sample resistance. Electrical resistivity *ρ* was calculated using the following formula:

$$\rho =R\frac{A}{l}$$

where *R*—electrical resistance of the sample, *l*—length of the sample, and *A*—cross-sectional area of the sample.

The three-point bend test was performed for evaluation of mechanical properties such as maximum bending stress *σ*_max_ and Young’s modulus *E* according to standard ASTM D7264/D7264M–21. The universal testing machine M500-50 CT (Testometric™, Rochdale, UK) equipped with a 5 N load cell was used for all tests. The samples of the size 35 mm × 10 mm × 4.3 mm (length × width × thickness) were cut and placed on a holder with 30 mm between support points. In a simplified, more visual manner, differences in mechanical strength were also observed by putting different weights (10 g, 50 g, and 100 g) on the center of the tested samples.

## 3. Results and Discussion

The electrochemical processes, which occurred on the surface of GF anode and cathode during electrodeposition in the abovementioned acidic electrolytes containing Pb(II) ions, are well known and thoroughly investigated [24], and can be described by the following reaction equations:

Cathodic processes:

$Pb_{\left(aq\right)}^{2+}+2e^{-}\to Pb_{\left(s\right)}$ the main process

$2H_{\left(aq\right)}^{+}+2e^{-}\;\to \;H_{2\left(g\right)}$ side process.

Anodic processes:

$Pb_{\left(aq\right)}^{2+}+2H_{2}O_{\left(l\right)}\to PbO_{2\left(s\right)}+4H_{\left(aq\right)}^{+}+2e^{-}$ the main process

$2H_{2}O_{\left(l\right)}\to 4H_{\left(aq\right)}^{+}+O_{2\left(g\right)}+4e^{-}$ side process.

Results after 1 h electrodeposition at 2.5 V are presented in Table 2. As seen from these data, an increase in the current efficiency of Pb and PbO_2_ electrodeposits was observed with ACT when additives were used. However, the current efficiency of Pb and PbO_2_ electrodeposits decreased when additives were present in NIT and MSA. Overall, MSA showed far greater electrodeposition current, hence the larger increase in the electrodes’ mass. This is important because a higher current would reduce the time required for a certain amount of material to be deposited, making the whole modification process faster. During electrodeposition experiments in ACT with ligninsulfonate, the color of electrolyte solution changed from initial bright yellow to brown, indicating side processes such as possible PbO_2_ breaking off and forming colloidal particles [25]. This darkening was not observed when lignosulfonate was used with NIT or MSA. During electrodeposition from MSA-T, the clear solution turned slightly yellow, whereas NIT-T and ACT-T remained clear. Triton X-100 can be electrochemically oxidized using PbO_2_ electrodes when greater than 10 mA cm^−2^ anodic current density is used [26].

**Figure 3:**
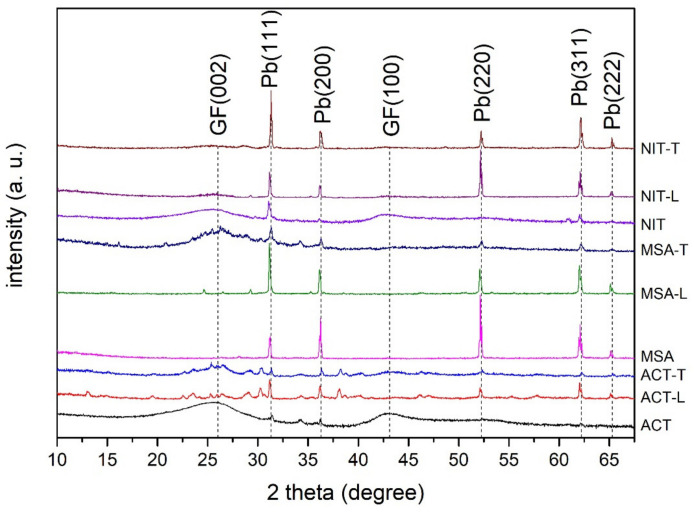
XRD of GF + Pb samples produced in various electrolytes.

XRD analysis revealed that GF + Pb samples had peaks corresponding to metallic lead (Figure 3). Samples with the highest mass increase (MSA, MSA-L, NIT-T, NIT-L) lacked the wide peaks at around 25.8° and 42.7°, corresponding to GF, indicating good coverage of GF fibers with Pb. Miniscule peaks associated with metallic Pb were present in XRD spectra of samples modified in ACT, ACT-L, NIT, MSA-T, and clear broad peaks of graphite indicated very poor coverage of GF fibers.

**Figure 4:**
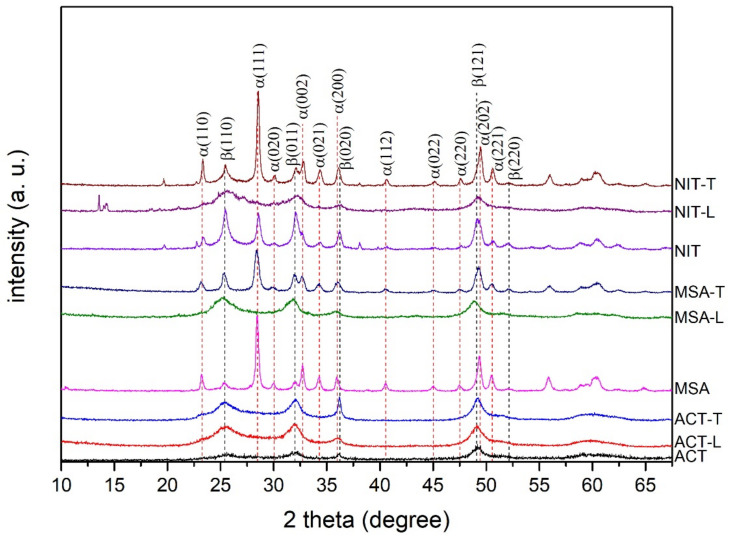
XRD of GF + PbO_2_ samples produced in various electrolytes.

XRD graphs of GF + PbO_2_ samples modified in NIT and MSA without additives and with Triton X-100 showed mostly a mix of α- and β-lead(IV) oxides (Figure 4). However, when ligninsulfonate was used as an additive during electrodeposition from NIT and MSA, the XRD curves had significantly wider peaks, suggesting a more amorphous phase and lack significant peaks associated with α-lead(IV) oxide. Therefore, it can be assumed that ligninsulfonate promotes amorphous crystal growth and shift the equilibrium toward the formation of primarily β-lead(IV) oxide. This phase is more desirable for electrochemical applications [27]. Furthermore, all samples modified in ACT showed similar patterns to samples modified in NIT-L or MSA-L with wide β-lead(IV) oxide peaks. Since no peaks of graphite were observed in any of the samples, the coverage of the outside GF fibers with PbO_2_ was considered adequate.

**Figure 5:**
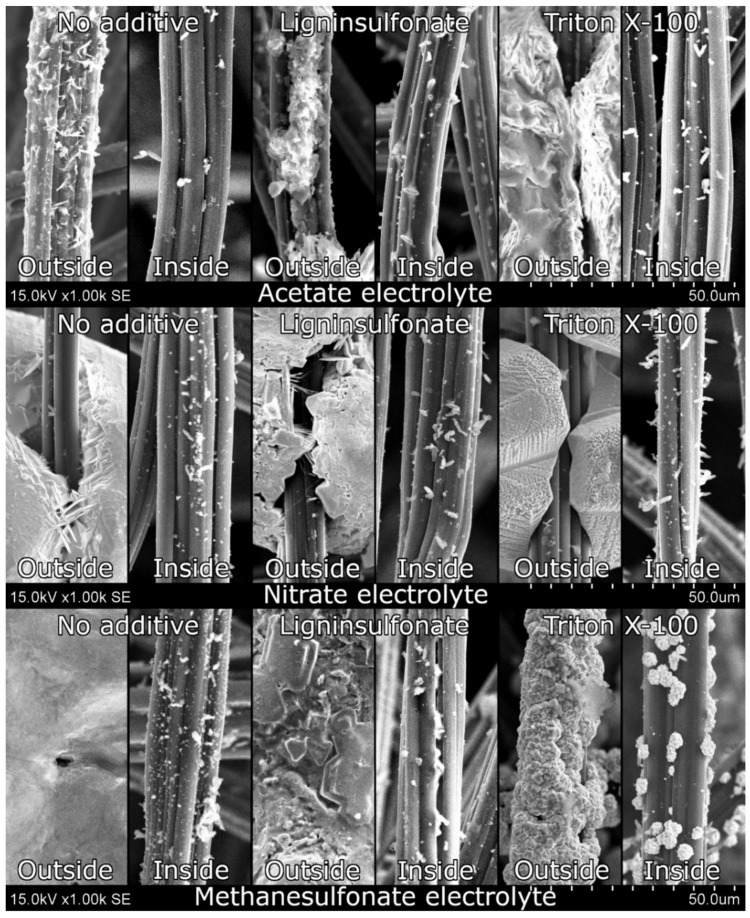
SEM images of samples polarized negatively during electrodeposition at 1000× magnification. The top, the middle, and the bottom rows show samples modified in acetate, nitrate, and methanesulfonate electrolytes, respectively. The left, the middle, and the right columns show samples modified without additives, with sodium ligninsulfonate, and with Triton X-100, respectively.

The most important quality factor of electrodeposition on GF is the distribution of deposited material throughout the thickness of GF, which was evaluated using SEM images (Figure 5). During electrodeposition from ACT on a negatively polarized electrode, crystals seemed to form smaller, more amorphous structures even on the outside of the GF. The internal fibers seemed to be poorly coated with small specs of lead crystals. Additives in this electrolyte did not seem to increase material dispersion through the thickness of the sample, whereas deposited material from NIT had the tendency to form sparse large crystals on the outermost fibers and very few small dendrites on the innermost fibers. Additives did not seem to change the size and shape of the deposited crystals, but the large crystal surface was changed, which was especially obvious with a ripple effect when Triton X-100 was used. The largest mass increase was observed when MSA was used, which was also shown in the SEM images where significantly more material on the fibers was observed than on the samples modified in ACT or NIT. Pb crystals were much larger than in the case of other electrolytes and almost completely covered the exterior fibers. Ligninsulfonate and Triton X-100 additives reduced the deposited crystal size and allowed them to spread even more on the fibers, even further increasing the coverage. Although electrodeposition results on the surface were satisfactory, the central fibers were still covered poorly. More lead crystals were observed on the innermost fibers than on samples modified in ACT or NIT, but this was still far from ideal coverage. Overall, Pb proved to be a very difficult material to electrodeposit on a 3D material such as GF.

**Figure 6:**
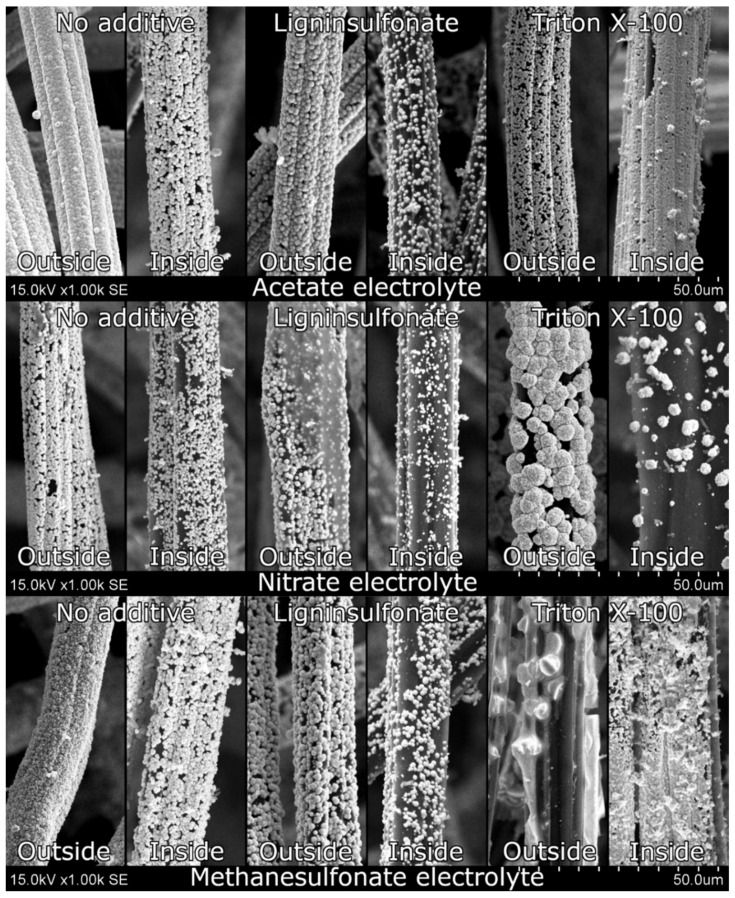
SEM images of samples polarized positively during electrodeposition at 1000× magnification. The top, the middle, and the bottom rows show samples modified in acetate, nitrate, and methanesulfonate electrolytes, respectively. The left, the middle, and the right columns show samples modified without additives, with sodium ligninsulfonate, and with Triton X-100, respectively.

GF + PbO_2_ samples were also analyzed using SEM images (Figure 6). Samples modified in ACT without additives had excellent GF fiber coverage with almost no gaps on the outside filaments of the GF but had some gaps in between the PbO_2_ crystals. Ligninsulfonate additive seemed to reduce the crystallinity of the deposited material. The increased amount and size of the gaps between crystals were probably due to lower deposited mass. Triton X-100 reduced the crystal particle size significantly but retained crystallinity. Furthermore, a completely different tendency was observed where more material was deposited on the inside fibers. Samples modified in the NIT electrolyte showed a decent coverage of the GF fibers with less coating on the innermost fibers. Ligninsulfonate once again decreased the crystallinity of the deposited material. Since the current efficiency was also lower during this electrodeposition, the observed material amount on the fibers was also significantly lower. Triton X-100 additive resulted in sphere-shaped crystal clusters, leaving large gaps between them. Furthermore, material deposited on the innermost fibers was reduced greatly. Finally, samples modified in MSA without additives showed a continuous layer of PbO_2_ formed from small crystals on the outside fiber, whereas inside fibers were covered with larger crystals with some gaps between them. Ligninsulfonate changed the deposited material, which was now formed from smooth surface spheres, and the total coverage was also smaller because of lower current efficiency. Triton X-100 completely changed the morphology to the one unseen on the samples before. All previous samples showed a layer of deposited material from small crystals, but the MSA-T sample displayed a continuous amorphous layer on the outside with some fractures throughout. Observed internal fiber coverage was very chaotic with varying size crystals and voids.

**Figure 7:**
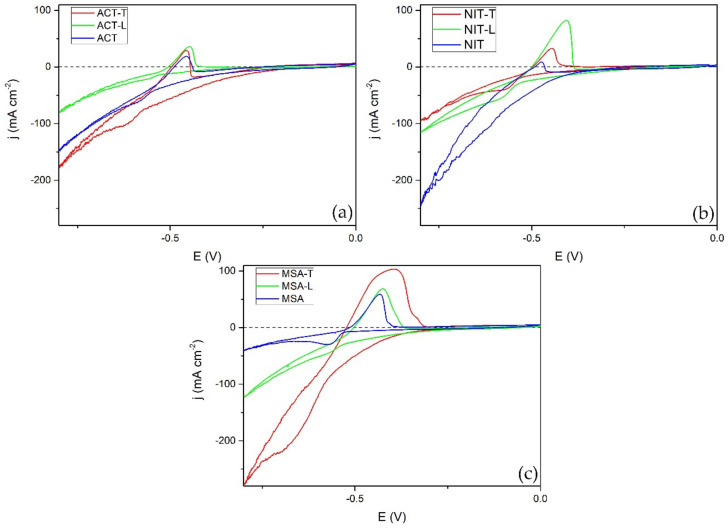
CV of GF + Pb samples produced in acetate (**a**), nitrate (**b**), and methanesulfonate (**c**) electrolytes using ligninsulfonate, Triton X-100, or without additives. Experiments were carried out in a 38% sulfuric acid electrolyte from −0.8 to 0.0 V vs. Ag/AgCl with a scan rate of 5 mV s^−1^.

The GF + Pb samples were analyzed using cyclic voltammetry in the range from −0.8 to 0.0 V vs. Ag/AgCl to determine whether they function as a lead electrode (Figure 7). In all cases, peaks corresponding to the conversion of metallic lead to lead(II) sulfate (at around −0.6 V) and the opposite reaction (at around −0.45 V) were observed. However, when either electrolyte additive was used, the peaks of cathodic and anodic current densities were more pronounced. Overall, samples that were modified using MSA showed the highest peak currents, whereas samples modified using ACT showed the smallest peaks. This correlates to mass increase on the GF substrate during electrodeposition. This means that using MSA could significantly reduce the total electrodeposition time required to cover GF fibers compared to NIT or ACT.

**Figure 8:**
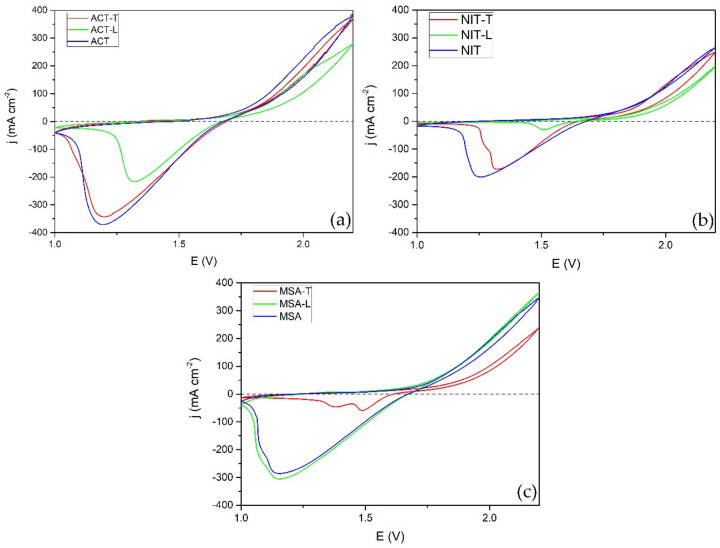
CV of GF + PbO_2_ samples produced in acetate (**a**), nitrate (**b**), and methanesulfonate (**c**) electrolytes using ligninsulfonate Triton X-100 or without additives. Experiments were carried out in a 38% sulfuric acid electrolyte from 1.0 to 2.2 V vs. Ag/AgCl with a scan rate of 5 mV s^−1^.

GF + PbO_2_ samples were also analyzed using cyclic voltammetry (Figure 8). In this case, the opposite tendency was observed. Firstly, samples modified in ACT showed the highest peak of lead(IV) oxide reduction to lead(II) sulfate when no additive or Triton X-100 was used, but the peak was significantly smaller when ligninsulfonate was used. By contrast, samples produced in MSA showed the second highest peaks in comparison with the sample modified in MSA-T, which showed a small double reduction peak. Secondly, composite electrodes produced in NIT showed peaks of modest intensity and when ligninsulfonate was used, the reduction peak was minuscule. Lastly, when electrodeposition was carried out using electrolyte additives, the process peaks were comparable or smaller. This correlates with lower current efficiencies discussed previously. The values of the peak current densities and potentials are listed in Table 3.

**Figure 9:**
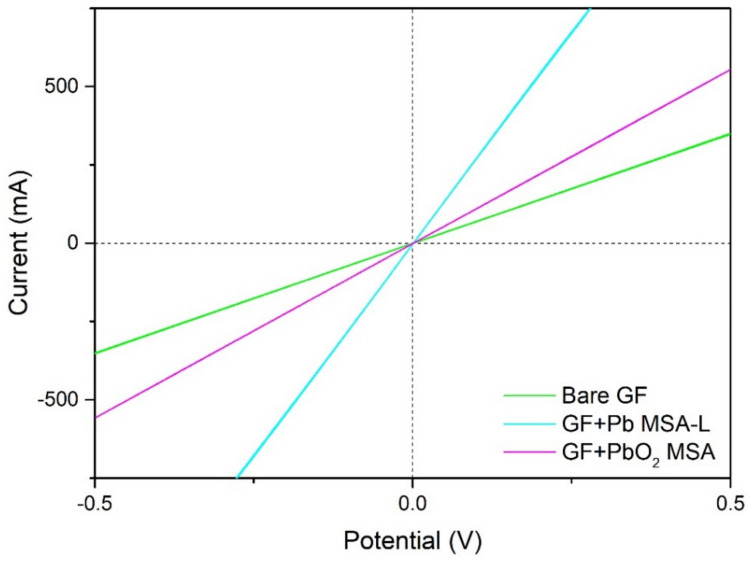
Linear voltage sweep at 20 mV s^−1^ results for bare GF, GF + Pb, and GF + PbO_2_.

Specific resistivity of bare GF and samples electrochemically modified in all electrolytes was measured by recording data while applying a linear sweep technique. As seen in Figure 9, the presented raw data (sample + clamps) from the experiment showed that both bare GF and modified composite samples, which demonstrated the best electrical conductivity, had a linear current response to changing voltage, and they behaved as conventional conductors, i.e., metals.

The calculated results are presented in Table 4. Overall, samples modified with metallic lead showed a decrease in resistivity with a maximum of more than fivefold decrease when GF was modified with metallic lead in MSA-L electrolyte. However, a slight increase in resistivity was observed when samples were modified in ACT-L and NIT-L. All GF + PbO_2_ samples showed a varying decrease in resistivity with an exception of samples modified in MSA-L. The resistivity of modified GF depends on how well the deposited material covers the fibers in a continuous layer, how many connections between adjacent fibers are formed, and the total loading of deposited material on the GF.

**Figure 10:**
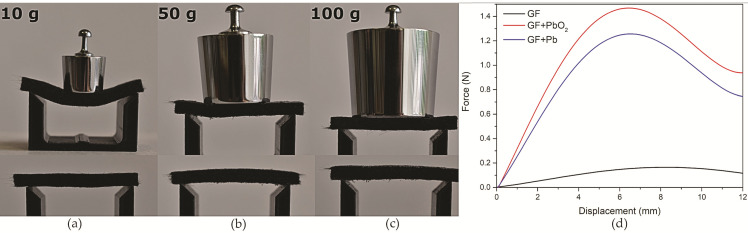
Visual bend test demonstration of bare GF (**a**), GF + Pb (**b**), and GF + PbO_2_ (**c**) samples modified in MSA-L and displacement–force curves of bending test for the same samples (**d**).

Mechanical strength test showed that unmodified GF sample (4.3 mm × 10 mm thickness × mm width) reached a maximum bending stress *σ*_max_ = 0.041 MPa, which was enough to hold its own weight when 30 mm distance was between supports but was a very flexible material even at light loads (Figure 10). However, after Pb was electrodeposited on the GF in MSA-L electrolyte, its mechanical strength increased significantly (*σ*_max_ = 0.303 MPa) and showed bending only at a defective site at 50 g and greater loads during a visual demonstration. Even greater strength was observed with the sample modified with PbO_2_, which showed no significant deformation even at 100 g load during the visual test and reached maximum bending stress of 0.361 MPa, although deposited mass on this sample was lower. This is most likely because PbO_2_ is a much stronger material than Pb. The obtained results demonstrated that the mechanical strength of both produced composite electrodes (GF + Pb and GF + PbO_2_) considerably increased, compared with that of bare GF and they provided sufficient mechanical strength to be used in LAB as a current collector.

## 4. Conclusions

The results of the investigation of electrochemical modification of graphite felt in acetate, nitrate, and methanesulfonate electrolytes, without or with ligninsulfonate or Triton X-100 additives, revealed that the best result for deposition of Pb was accomplished by using methanesulfonate electrolyte with ligninsulfonate. However, poor Pb coverage on the innermost graphite felt fibers was still observed, while acetate electrolyte without additives provided the best coating of PbO_2_ on graphite felt, and the dispersion of the particles throughout the depth of graphite felt was excellent. β-PbO_2_ was deposited when acetate electrolyte was used, whereas a mix of α and β phases was observed when nitrate or methanesulfonate electrolyte was used. When a ligninsulfonate additive was added to nitrate or methanesulfonate electrolyte, it shifted the phase of PbO_2_ to β but reduced the crystallinity.

Electrochemical analysis of the produced composite electrodes showed that they acted as a lead or lead(IV) oxide when subjected to appropriate potential changes in sulfuric acid electrolyte. The most electrochemically active electrodes were produced in methanesulfonate and acetate electrodes for Pb and PbO_2_, respectively. Furthermore, a tendency was observed that Pb deposited from methanesulfonate electrolytes with Triton X-100 additive was more electrochemically reactive; however, an opposite trend was seen for PbO_2_, in the case of which Triton X-100 additive significantly reduced the electrochemical activity.

Some physical properties of the produced composite electrodes were also evaluated. A varied decrease in electrical resistivity was observed after graphite felt was modified with Pb or PbO_2_ with a couple of exceptions. More than a fivefold decrease in electrical resistivity was observed when GF was modified with Pb in methanesulfonate electrolyte with ligninsulfonate additive. Both Pb and PbO_2_ provided significant additional mechanical strength, which should provide sufficient mechanical strength for these composite electrodes to be used in lead-acid batteries.

Composite graphite felt and lead(IV) oxide samples should be considered as potential candidates for replacing the traditional lead grids due to the excellent distribution of the deposited material throughout the depth of the graphite felt. However, additional studies should be carried out to determine the optimal loading of the deposited material for the best balance between lower electrical resistivity, higher mechanical strength, and lower mass.

## Figures and Tables

**Table 1:** Composition of electrolytes used for electrochemical deposition on GF.

Electrolyte	Component 1	Component 2	Component 3	pH
ACT	Pb(CH_3_COO)_2_ 0.5 M	CH_3_COOH 1 M	CH_3_COONH_4_ 1 M	4.5 ± 0.5
NIT	Pb(NO_3_)_2_ 0.5 M	HNO_3_ 0.1 M	-	0.0 ± 0.5
MSA	Pb(CH_3_SO_3_)_2_ 0.5 M	CH_3_SO_3_H 0.5 M	-	0.0 ± 0.5

**Table 2:** Electrodeposition results after 1 h.

Additive		None		Ligninsulfonate		Triton	
Electrolyte	Electrode	Deposited Mass, g g^−1^ GF	Current Efficiency, %	Deposited Mass, g g^−1^ GF	Current Efficiency, %	Deposited Mass, g g^−1^ GF	Current Efficiency, %
MSA	+ *	1.001	95.7	0.493	55.7	0.587	69.6
	-	1.446	79.8	1.010	65.8	0.840	57.5
ACT	+ *	0.482	94.7	0.411	90.0	0.435	99.8
	-	0.472	53.5	0.617	78.1	0.622	82.3
NIT	+ *	0.505	92.1	0.233	45.3	0.197	34.1
	-	0.467	49.1	0.537	60.3	0.384	38.4

*—for these electrodes (both anodes) the average values of deposited mass and current efficiency were calculated.

**Table 3:** Redox peak parameters of GF-Pb and GF-PbO_2_ samples in 38% sulfuric acid electrolyte during CV tests.

Sample		GF + Pb						GF + PbO_2_	
	Additive	*E*_pa_, mV	*E*_pc_, mV	*j*_ac_, mA cm^−2^	*j*_pc_, mA cm^−2^	Δ*E*, mV	*j*_pc_/*j*_pa_	*E*_pc_, mV	*j*_pc_, mA cm^−2^
ACT	No additive	−454	−587	18.9	60.6	133	0.31	1192	371.0
	Ligninsulfonate	−449	−555	35.9	20.5	106	1.75	1322	216.0
	Triton X-100	−457	−609	29.3	97.3	152	0.30	1196	343.0
MSA	No additive	−430	−534	58.1	28.7	104	2.02	1156	246.2
	Ligninsulfonate	−424	−566	68.3	43.5	142	1.57	1140	258.3
	Triton X-100	−398	−665	103.3	197.7	267	0.52	1477	58.6
NIT	No additive	−470	- *	12.7	- *	- *	- *	1286	207.5
	Ligninsulfonate	−405	−581	81.9	54.4	176	1.51	1511	30.0
	Triton X-100	−445	−566	32.8	38.2	121	0.86	1336	183.7

*—cathodic peak was not observed.

**Table 4:** Resistivity measurement results of bare GF, GF + Pb, and GF + PbO_2_ samples modified in various electrolytes with and without additives.

Sample	Electrical Resistivity, ×10^3^ Ω m	Change, %	Electrical Resistivity, ×10^3^ Ω m	Change, %
Bare GF	26.0			
	Pb		PbO_2_	
ACT	22.3	−14.4	17.3	−33.6
ACT-L	52.5	+101.7	23.0	−11.8
ACT-T	22.7	−12.9	18.6	−28.4
NIT	19.9	−23.6	15.6	−40.1
NIT-L	47.3	+81.9	24.3	−6.7
NIT-T	23.7	−8.9	23.8	−8.5
MSA	14.5	−44.2	15.3	−41.0
MSA-L	4.7	−81.9	34.9	+34.0
MSA-T	7.2	−72.5	23.7	−8.9

## Data Availability

The data presented in this study are available on request from the corresponding author.

## Abbreviations

LAB	Lead-acid batterie(s)
GF	Graphite felt
NIT	Nitrate electrolyte
ACT	Acetate electrolyte
MSA	Methanesulfonate electrolyte
CV	Cyclic voltammetry
L	Ligninsulfonate additive
T	Triton X-100 additive
GF + Pb	GF sample modified with Pb (polarized negatively during modification)
GF + PbO_2_	GF sample modified with PbO_2_ (polarized positively during modification)
